# Book Review

**Published:** 1907-03

**Authors:** 


**An Introduction to the Study of Colour Phenomena.** By JOSEPH W. LOVIBOND. Pp. 48. London: E. and F. Spon. 1905.—This small volume is apparently an extension of the author's previous work on *The Measurement of Light and Colour Sensations*. To the former material has been added a new colour theory and a more elaborate system of colour nomenclature. With the description of the apparatus designed for measuring and recording colour sensation in quantitative terms the interest which the writer commands is exhausted. By the combination of single sensation colours (red, yellow and blue)

in thin glass plates, the tint of which is arbitrarily regarded as a standard unit, varying depths of a particular colour and differing shades may be imitated. As an instance we may quote, two blue plus two red develop two units of violet; that is to say, white light transmitted through two thicknesses of the unit blue glass and the unit red produces two units of violet colour for purposes of merely empirical comparison; this is interesting, and may be useful. No scientific basis for his standard is suggested by Mr. Lovibond. As a practical method it is attractive in its simplicity. The author's analytical colour charts form an excellent means of recording in a simple form the results of investigations with his "tintometer," and the application of these charts to the hæmoglobinometer earned for him a medal at the St. Louis Exposition of 1904. When, however, the new colour theory is expounded we are at a loss to see any reason for substituting a clumsy "six-ray" theory for the accepted "three-ray," and the writer does not bring forward any arguments that make good his claim to the discovery of a simpler or more satisfactory explanation of colour phenomena. Nor do numerous highly-coloured, solid, geometrical figures make up for a total absence of all references to rates of vibration and wave-lengths in the production of varying colour sensations.

**Studies in Blood Pressure: Physiological and Clinical.** By George Oliver, M.D. Pp. 151. London: H. K. Lewis. 1906.—These two lectures, on the physiological and clinical aspects of blood-pressure measurement, are likely to be of much service at a time when clinical hæmomanometric methods are engaging so much attention. This little book may be regarded as a text-book by one who has been the pioneer on the subject of hæmomanometry, and whose clinical observations are most trustworthy.

**Manual of Medicine.** By T. K. Monro, M.D. Second Edition. Pp. xxii., 1022. London: Baillière, Tindall & Cox. 1906.—The second edition of this manual has been subjected to a thorough revision throughout, and a good deal of new matter added, bringing the volume thoroughly up to date. Additional illustrations have also been introduced. Although the work is longer than its predecessor, it is not unduly voluminous, and we have failed to find any omissions of importance.

**The Treatment of Diseases of the Digestive System.** By Robert Saundby, M.D. Pp. viii., 133. London: Charles Griffin & Co., Ltd. 1906.—The most essential part of this booklet is the introduction, an essay of forty-one pages on the personal experience and views of a physician of large and mature experience on a subject on which many volumes have been and may still be written. The subsequent chapters on diseases of the œsophagus, stomach, intestine and rectum, are such as might be culled from any text-book. A short essay on symptomatic diseases, and a long list of formulæ of the ordinary type complete the volume, which is not likely to enhance the reputation of its already distinguished author.

**Minor Maladies and their Treatment.** By LEONARD WILLIAMS, M.D. Pp. vii., 383. London: Baillière, Tindall & Cox. 1906.—This volume of 383 pages is a reprint from various sources of lectures given at the Medical Graduates' College and Polyclinic. They must have served a very useful purpose, and are quite worthy of reproduction in book form, inasmuch as much of the contents can only be learned by prolonged experience, and is not to be found in the text-books. The subjects are of every-day interest and utility, ranging from the common catarrh up to insanity, and many well-tried formulæ are scattered through the text. These are at the present time of increasing use, inasmuch as few students learn the elements of prescribing, and are too prone to adopt the unscientific combinations of the manufacturing druggist, for whose benefit they are commonly made rather than for the patient.

**Uric Acid: Chemistry, Physiology and Pathology.** By FRANCIS H. McCRUDDEN. New York: Hœber. 1906.—To an inquiry as to the time of publication of his masterpiece, an author humorously replied, "The head is on the perineum." Such a phrase could never have been applied to this book. True, it exhibits the traces of tedious labour, but there is not a spark of life within its pages, there is nothing which stimulates the mind or widens the imagination. It is a machine-made book pure and simple. To borrow a phrase from the greener isle, "It was killed at conception." For the preface, with engaging frankness, states that the publication is made because there is no complete and reliable account of the metabolism of uric acid to be found in one place. Completeness, reliability—life is short—are to compensate for vivacity, purpose, interest. But such a programme is too extensive for even machine-made books. Were one to be hypercritical, several errors in chronology and omissions of work could be pointed out. It is not necessary to do this, for with a book of this kind it is impossible to approach perfection. Do not, however, take our diatribe too seriously. Provided that it is necessary for anyone to refer to the uric acid literature, then this book will save much searching. In the States it even might assist spelling, and that is saying much for an American book. But as the practitioner does not care to search for literature since he receives the *Uric Acid Monthly*, without cost or intermission, and prefers to confine his spelling errors to his prescriptions, the book then hardly appeals to him. To whom then shall it be recommended? The physiologist, the pathologist, the librarian, the book lover, the collector for posterity? All these—poor souls in more senses than one—will rejoice over this triple essence of uric acid research and conjecture. Possession will mean satisfaction—and, after all, what better does life provide? In this way the author may not have laboured in vain.

**On Retro-peritoneal Hernia.** By B. G. A. Moynihan, M.S., F.R.C.S. Second Edition. Revised and in part rewritten by the Author and J. F. Dobson, M.S., F.R.C.S. Pp. vi., 195. London: Baillière, Tindall & Cox. 1906.—Since the first edition of this work was published in 1899 a good deal of anatomical work has been done in this subject, which has been duly incorporated in the present edition. It is fully illustrated, and there is a complete bibliography of the different varieties of hernia met with in this region. The book is certainly one of the best accounts which we have in English on this serious affection.

**The Extra Pharmacopœia of Martindale and Westcott.** Revised by W. Harrison Martindale and W. Wynn Westcott, M.B. Twelfth Edition. Pp. xxx., 1045. London: H. K. Lewis. 1906.—Nearly 250 pages of new matter have been added, and every care has been taken to make the work as complete as possible. The authors urge rightly that it is time that the practice which has grown up of burdening the prescriber with metric equivalents to grains, drachms, and ounces should be dropped in favour of metric terms only, which involve far less mental calculation, and hence involve very much less likelihood of error. They also urge that it would be of general advantage if the English term minim were abandoned: a drop might be considered as $\frac{1}{20}$ of a cubic centimetre, or about $\frac{7}{8}$ of a minim.

**The Care of Children.** By Robert J. Blackham, Capt. R.A.M.C. Revised and enlarged edition. Pp. xi., 84. London: Scientific Press, Ltd. 1906.—Truly the times have changed from those when Kingsley could describe the army doctor as one who thought a baby's inside much the same as that of a Scotch guardsman. This excellent little book is written with a view of helping to educate mothers and nurses in the simple but essential principles of infant hygiene, by one who has been impressed with the extremely disastrous results which are occurring all over this country owing to the well-nigh universal ignorance of the populace concerning this subject. Captain Blackham, who writes from the Military Families' Hospital, Devonport, has evidently a very practical knowledge of his subject. He does not preach any new doctrines, but merely sets down in clear and convenient form the accepted teaching on infant feeding, clothing and general hygiene. The book seems sound in every respect, and carefully prepared to meet a very real want. We trust it may have a wide circulation among those for whose use it has been written, and may help to diminish the terrible infant mortality rate about which so much is talked, though comparatively little is done to diminish it.

